# Individualized Prediction of Colorectal Cancer Metastasis Using a Radiogenomics Approach

**DOI:** 10.3389/fonc.2021.620945

**Published:** 2021-04-28

**Authors:** Qin Liu, Jie Li, Lin Xu, Jiasi Wang, Zhaoping Zeng, Jiangping Fu, Xuan Huang, Yanpeng Chu, Jing Wang, Hong-Yu Zhang, Fanxin Zeng

**Affiliations:** ^1^Department of Clinical Research Center, Dazhou Central Hospital, Dazhou, China; ^2^Department of Radiology, Dazhou Central Hospital, Dazhou, China; ^3^Department of Clinical Laboratory, Dazhou Central Hospital, Dazhou, China; ^4^Department of Ophthalmology, Medical Research Center, Beijing Chaoyang Hospital, Capital Medical University, Beijing, China; ^5^Department of Clinical Laboratory, Beijing Chaoyang Hospital, Capital Medical University, Beijing, China; ^6^Hubei Key Laboratory of Agricultural Bioinformatics, College of Informatics, Huazhong Agricultural University, Wuhan, China; ^7^School of Medical and Life Sciences, Chengdu University of Traditional Chinese Medicine, Chengdu, China

**Keywords:** colorectal cancer, metastasis, radiomics, genomics, carbohydrate antigen 19-9

## Abstract

**Objectives:** To evaluate whether incorporating the radiomics, genomics, and clinical features allows prediction of metastasis in colorectal cancer (CRC) and to develop a preoperative nomogram for predicting metastasis.

**Methods:** We retrospectively analyzed radiomics features of computed tomography (CT) images in 134 patients (62 in the primary cohort, 28 in the validation cohort, and 44 in the independent-test cohort) clinicopathologically diagnosed with CRC at Dazhou Central Hospital from February 2018 to October 2019. Tumor tissues were collected from all patients for RNA sequencing, and clinical data were obtained from medical records. A total of 854 radiomics features were extracted from enhanced venous-phase CT of CRC. Least absolute shrinkage and selection operator regression analysis was utilized for data dimension reduction, feature screen, and radiomics signature development. Multivariable logistic regression analysis was performed to build a multiscale predicting model incorporating the radiomics, genomics, and clinical features. The receiver operating characteristic curve, calibration curve, and decision curve were conducted to evaluate the performance of the nomogram.

**Results:** The radiomics signature based on 16 selected radiomics features showed good performance in metastasis assessment in both primary [area under the curve (AUC) = 0.945, 95% confidence interval (CI) 0.892–0.998] and validation cohorts (AUC = 0.754, 95% CI 0.570–0.938). The multiscale nomogram model contained radiomics features signatures, four-gene expression related to cell cycle pathway, and CA 19-9 level. The multiscale model showed good discrimination performance in the primary cohort (AUC = 0.981, 95% CI 0.953–1.000), the validation cohort (AUC = 0.822, 95% CI 0.635–1.000), and the independent-test cohort (AUC = 0.752, 95% CI 0.608–0.896) and good calibration. Decision curve analysis confirmed the clinical application value of the multiscale model.

**Conclusion:** This study presented a multiscale model that incorporated the radiological eigenvalues, genomics features, and CA 19-9, which could be conveniently utilized to facilitate the individualized preoperatively assessing metastasis in CRC patients.

## Introduction

Colorectal cancer (CRC) is a common malignant tumor, for which the incidence of men is usually higher than women. In 2012, more than 690,000 people died of CRC worldwide (1). At present, the main treatment of CRC is surgical resection, supplemented by chemotherapy or radiotherapy. However, more than 20% of patients are still life-threatening because of disease metastasis (2). Accurate prediction of tumor metastasis is of great significance for the individualized treatment and prognosis of patients with CRC (3). Computed tomography (CT), a non-invasive imaging tool, has been used for screening and preoperative evaluation of various cancers (4–7). In CRC patients, the venous phase of enhanced CT scan shows a stronger resolution and good response to the size and scope of tumors (8). However, CT has limited specificity in the differential diagnosis of distal metastasis, metastatic lymph nodes, and lesions with smaller diameter (9, 10). Therefore, we need to find more specific method to distinguish CRC metastasis.

Quantitative high-throughput analysis of a large number of image data obtains radiomics eigenvalues, which allows for more in-depth mining of CT data (11, 12). Radiological eigenvalues can be used as predictive biomarkers to diagnose, treat, and evaluate the disease. Several investigators have shown that the radiomics is closely related to tumor stages and grades (13, 14), and the combined analysis of a set of biomarkers is more practical than single indicators (15). CA-19-9, known as a tumor marker, has been reported to be significantly increased in gastrointestinal cancers (16, 17). It is also used to combine with other clinical indicators evaluating the metastasis of CRC (18). A study has reported that combined radiomics signatures and clinical risk factors such as carcinoembryonic antigen (CEA) are valuable to evaluate the lymph node metastasis in CRC patients (19). Although these features obtained by radiomics that are combined with clinical indicators have been used as useful predictors to predict indeterminate pulmonary nodules and lymph node metastasis in CRC (19, 20), these biomarkers often focus on one or a few aspects of the progression in CRC. Few studies have combined more accurate predictors from different dimensions such as genomics, which may provide more available risk assessment information. In recent years, genomics plays an important role in the diagnosis and treatment of cancers (21). Some studies have explored the metastasis and precise treatment of CRC through genomics analysis (22, 23). The abnormal cell cycle is a typical characteristic of tumor (24). *CDKN2A, TP53, ATM*, and *MYC* play an important role in cell cycle, which regulates the cell growth and proliferation (25, 26). However, an optimal approach, combined with more dimensions, is needed to improve the prediction model performance.

Therefore, the aim of this study was to develop and validate a multiscale model by combining radiomics signatures, genomics features, and clinical risk factors for evaluation of preoperative metastasis in CRC patients.

## Materials and Methods

### Patients

The study was approved by the ethics committee of Dazhou Central Hospital (IRB00000003-17003) and obtained informed consent from patients. The study retrospectively reviewed 267 patients clinicopathologically diagnosed with CRC from February 2018 to March 2019 as primary and validation cohorts and reviewed 116 patients from April 2019 to October 2019 as independent-test cohort. The participants underwent surgical resection for therapeutic purposes. Finally, 90 patients were included in primary and validation cohorts. Independent-test cohort included 44 CRC patients. All CRC tissue samples were obtained intraoperatively for RNA sequencing and stored in liquid nitrogen. The clinical stage of tumors was according to tumor–node–metastasis staging system [American Joint Committee on Cancer, 8th edition, staging system]. The metastasis was defined as patients with pathologically diagnosed CRC metastasis after surgery or within 3 months after surgery.

The 90 participants were randomly divided into a primary cohort and a validation cohort at a ratio of ~7:3. In total, the primary cohort comprised 62 patients (38 males and 24 females, 24 metastasis and 38 non-metastasis patients). There were 28 patients in the validation cohort (16 males and 12 female, 11 metastasis, and 17 non-metastasis patients). In the independent-test cohort, there were 25 metastasis and 19 non-metastasis patients (29 males and 15 females). Clinical data, including age, gender, preoperative histological grade, and CA 19-9, were obtained from medical records.

**Figure 1:**
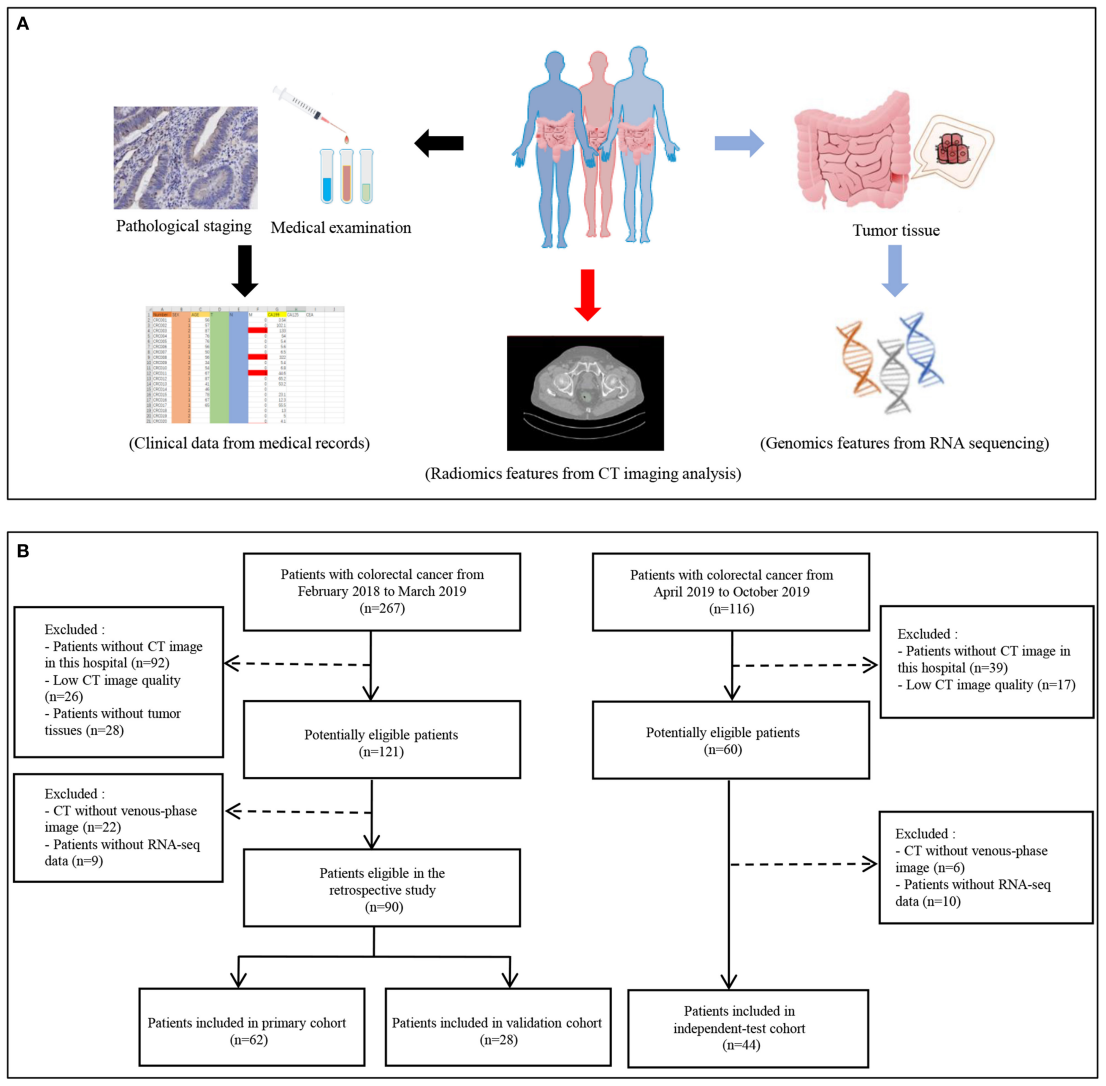
Flowchart of study. **(A)** The data collection pipeline in this study. **(B)** Flowchart of patients' inclusion.

Serum CA 19-9 levels of the patients were detected by CA 19-9 test kit (Roche Diagnostics Corp., Switzerland) with cobas e601 system at the initial hospitalization. The recommended normal range of CA 19-9 is 0–27 ng/mL. The flowchart of the study is shown in Figures 1A,B. Inclusion criteria for the study were as follows: (1) patients with baseline and 2-weeks complete enhanced CT examinations; (2) CT images were obtained with 1-mm thickness; (3) the patient diagnosed with CRC by pathology; and (4) age 18–75 years. Exclusion criteria were as follows: (1) patients with inflammatory diseases, including infection, ischemic heart disease, collagen disease, intestinal perforation, or obstruction; (2) patients with familial adenomatous polyposis or hereditary nonpolyposis colon cancer; and (3) patients, lacking CT images or with poor image quality, could not extract radiomics features.

### CT Image Acquisition and Analysis

The imaging ensemble data acquisition was performed on the abdominal CT enhanced scan using Siemens Definition AS 64-row CT. The acquisition parameters were as follows: 100 kV, CAREDose4D, 0.5-s rotation time, detector collimation: 128 × 0.6 mm, field of view 380 × 380 mm, matrix: 512 × 512; after routine CT plain scan, 60–80 mL iodophor contrast agent (320 mg/mL) was used at a speed of 2–3 mL/s and with a high-pressure syringe (Ulrich, Germany), and then 40 mL of normal saline was injected, after 23 s. Arterial scan, 60-s postvenous scan, delayed the scan after 120 s; delayed prolonged scan time according to the lesion, contrast-enhanced CT reconstruction with thickness 1 mm. Then, the CT image of the portal vein (thickness 1.0 mm) DICOM data was retrieved from CD Viewer (INFINITT, Seoul, South Korea). Two professional data readers used the 3D Slicer software (version 4.10.2) to delineate the tumor tissue under the guidance of a clinical imaging specialist for building a three-dimensional tumor tissue (27, 28). Finally, the eigenvalue results were derived from the SlicerRadiomics plug-in.

### Radiomics Signature Extraction

The primary cohort was used to establish the prediction models. The 10-fold cross-validation least absolute shrinkage and selection operator (LASSO) was used for selecting the most valuable radiomics features via minimum criteria. The radiomics score was calculated for each participant by a linear combination of screened features weighted by their respective LASSO coefficients.

### Genomics Feature Selection

Total RNA of tumor tissues collected from CRC patients was extracted by TRIZOL reagent (Takara Biomedical Technology, Beijing, China). Eligible total RNA was purified and fragmented for further sequencing library construction. Qualified sequencing libraries were sequenced by Illumina with sequencing strategy PE150. Clean reads were used for subsequent analysis. The differentially expressed genes between metastasis and non-metastasis groups were analyzed by DEseq2 package in R software. Protein–protein interaction network (https://string-db.org/) was applied to analyze the potential interaction.

### Construction and Performance of the Multiscale Nomogram

The prediction model was developed based on the selected radiomics features by using multivariable logistic regression analysis. The model was converted into prediction nomogram for providing clinicians with an easy-to-use tool to facilitate the individual probability of CRC preoperative metastasis. The predictive performance of the multivariate nomogram was assessed in the primary cohort and then verified in the validation cohort and independent-test cohort. The receiver operating characteristic curve (ROC) analysis was used as a performance indicator. In order to estimate the clinical usefulness of the prediction nomogram, the decision curve was conducted by calculating the net benefits for a range of threshold probabilities.

### Statistical Analysis

Statistical analysis was performed using R software [version R 3.6.1 for Windows (x64)] and IBM SPSS (version 20.0). The appropriate package and function to complete the corresponding statistical test were loaded. The “pROC” package (version 1.15.3) was used to plot ROC curves. The “glmnet” package (version 2.0–18) was used to finish the LASSO model. The “rms” package (version 5.1–3.1) was performed for calibration curve. The “rmda” package (version 1.6) was performed for decision curve. At inspection level, *P* < 0.05 is considered statistically significant. The differences of the basic clinical information between metastasis and non-metastasis groups were performed by *t-*test or χ^2^ test.

## Results

### Clinical Phenotype Data

The characteristics of patients in the primary cohort, validation cohort, and independent-test cohort are shown in Table 1, Supplementary Tables 1, 2. There was no difference in clinical data between the primary and validation cohorts (Supplementary Table 1). CA 19-9 showed a significant difference between metastasis and non-metastasis groups in the primary cohort (*P* < 0.05), which was then confirmed in the validation cohort (*P* < 0.05) (Table 1).

**Table 1:** Characteristics of patients in the primary and validation cohorts.

**Characteristic**	**Primary cohort**			**Validation cohort**		
	**Non-metastasis** **(*n =* 38)**	**Metastasis** **(*n =* 24)**	***P***	**Non-metastasis** **(*n =* 17)**	**Metastasis** **(*n =* 11)**	***P***
Age (mean ± SD), years	59.68 ± 10.84	58.46 ± 12.92	0.689	60.01 ± 12.24	61.64 ± 12.56	0.744
Sex, no. (%)			0.063			0.441
Male	27 (71.05)	11 (45.83)		11 (64.71)	5 (45.45)	
Female	11 (28.95)	13 (54.17)		6 (35.29)	6 (54.55)	
CA 19-9 level, no. (%)			0.008^*^			0.023^*^
Normal	31 (83.78)	11 (50.00)		14 (93.33)	5 (50.00)	
Abnormal	6 (16.22)	11 (50.00)		1 (6.67)	5 (50.00)	
Tumor stage, no. (%)			<0.001^*^			<0.001^*^
0	4 (10.53)	0 (0.00)		0 (0.00)	0 (0.00)	
I	18 (47.37)	0 (0.00)		7 (41.18)	0 (0.00)	
II	16 (42.11)	0 (0.00)		10 (58.82)	0 (0.00)	
III	0 (0.00)	20 (83.33)		0 (0.00)	6 (54.55)	
IV	0 (0.00)	4 (16.67)		0 (0.00)	5 (45.45)	
Tumor sites, no. (%)			0.077			0.254
Rectum	30 (78.95)	12 (50.00)		13 (76.47)	6 (54.54)	
Right colon	4 (10.53)	4 (16.67)		1 (5.88)	4 (36.36)	
Left colon	3 (7.89)	4 (16.67)		1 (5.88)	0 (0.00)	
Radiomics score (mean ± SD)	−3.71 ± 3.72	3.37 ± 5.17	<0.001^*^	−0.81 ± 4.35	4.15 ± 6.18	0.02^*^
Sigmoid colon	1 (2.63)	4 (16.67)		1 (5.88)	0 (0.00)	
Transverse colon	0 (0.00)	0 (0.00)		1 (5.88)	1 (9.10)	

*T test or χ^2^ test was used to compare characteristics differences between non-metastasis and metastasis groups in primary and validation cohorts*.

* *P < 0.05*.

*The upper reference limit value for CA 19-9 level was 27 ng/mL in clinic. Abnormal: CA 19-9 level >27 ng/mL; normal: CA 19-9 level ≤27 ng/mL*.

*SD, standard deviation; CA-19-9, carbohydrate antigen 19-9*.

### Radiomics Signature Building

**Figure 2:**
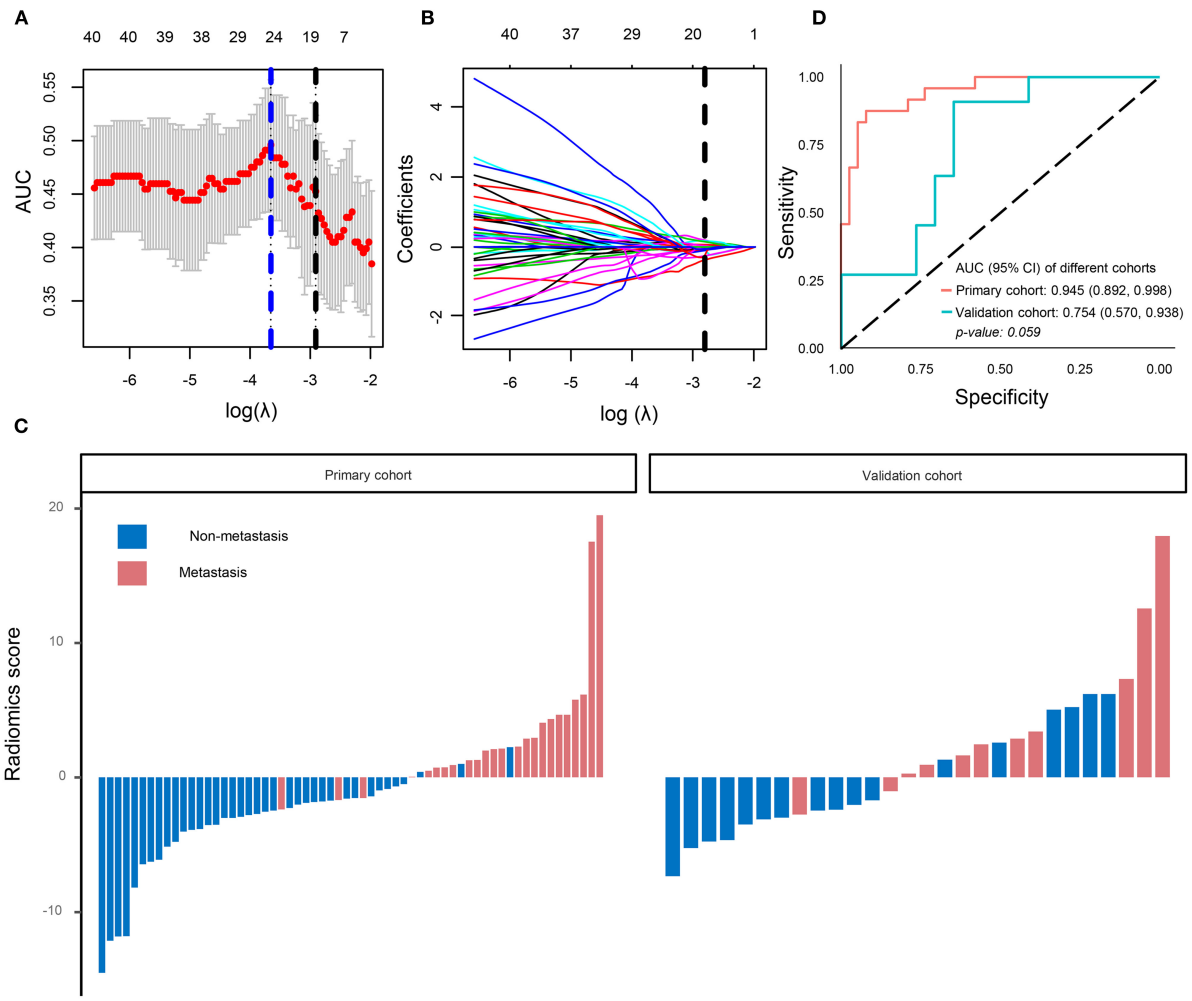
Radiomics signatures selection and model performance. **(A)** Tuning parameter (λ) was selected in the LASSO model by 10-fold cross-validation via minimum criteria. The area under the receiver operating characteristic curve was plotted versus log(λ). A log (λ) = −2.91 was chosen. **(B)** LASSO coefficient profiles of the 854 texture features. A coefficient profile plot was produced against the log (λ) sequence. Vertical line was plotted at the optimal λ value, which resulted in 16 non-zero coefficients. **(C)** The radiomics score for each patient in the primary (AUC) cohort (*n* = 62) and validation cohort (*n* = 28). **(D)** ROC curve of radiomics model in the primary cohort and validation cohort. LASSO, least absolute shrinkage and selection operator.

Using the LASSO regression model, 854 texture features were reduced to 16 potential predictors (ratio 53:1; Figures 2A,B). The radiology eigenvalues were calculated by radiomics score calculation formula (Supplementary File 1). The radiomics score was significantly different between metastasis and non-metastasis in the primary and validation cohorts (*P* < 0.05) (Table 1, Figure 2C). The area under curve (AUC) of the radiomics model reached 0.945 [95% confidence interval (CI) 0.892–0.998] in the primary cohort and 0.754 (95% CI 0.570–0.938) in the validation cohort, respectively (Figure 2D). Typical cases of non-metastasis and metastasis are presented in Supplementary Figure 2.

### Development and Validation of the Multiscale Model

**Figure 3:**
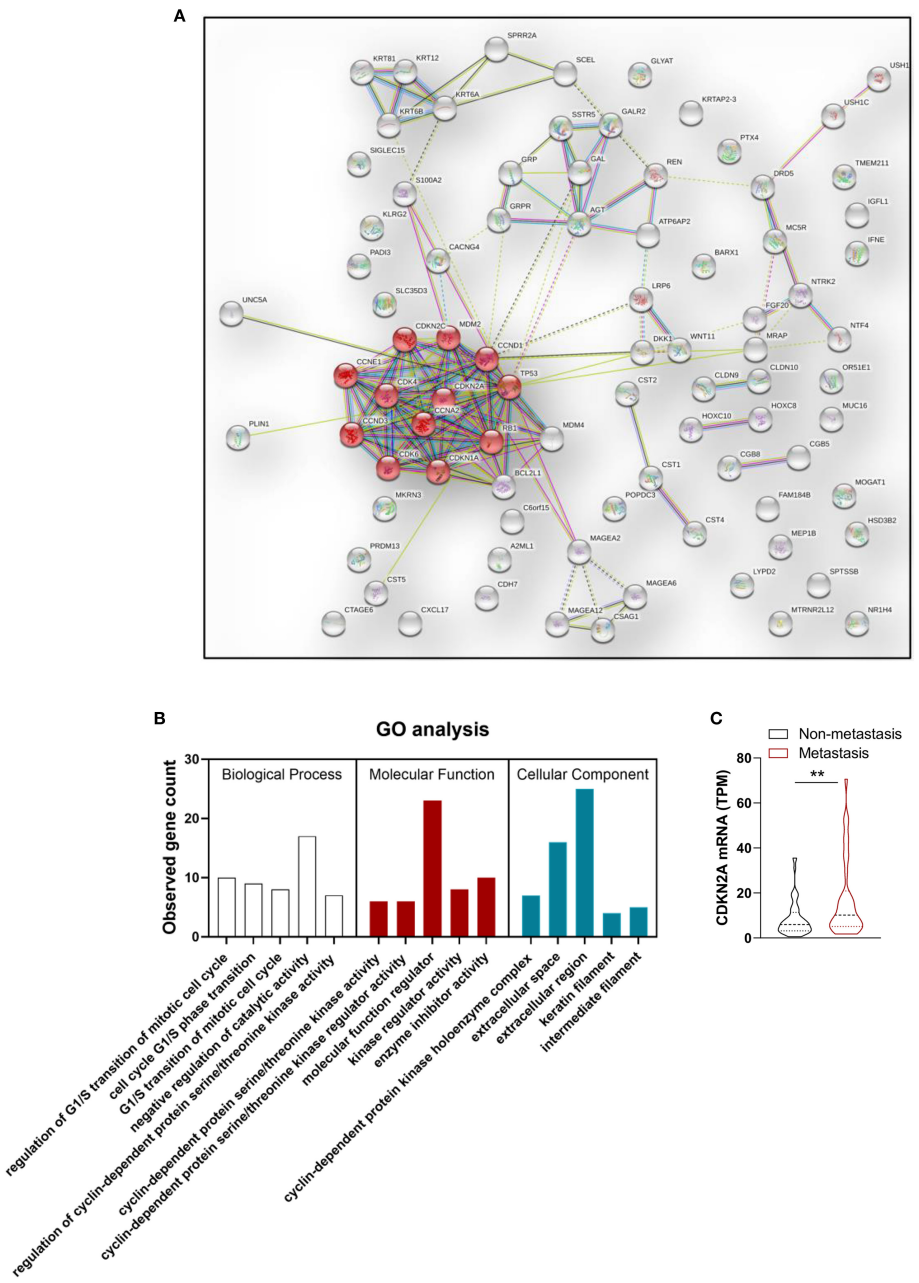
Genomics features selection. **(A)** Protein–protein interaction (PPI) network and **(B)** Gene Ontology (GO) enrichment analysis of differentially expressed genes between metastasis group and non-metastasis group. Red node represents proteins enriched in cell cycle pathway. **(C)** Statistical analysis of *CDKN2A* mRNA expression level in metastasis group and non-metastasis group. *n* = 90. ^**^P < 0.01.

**Figure 4:**
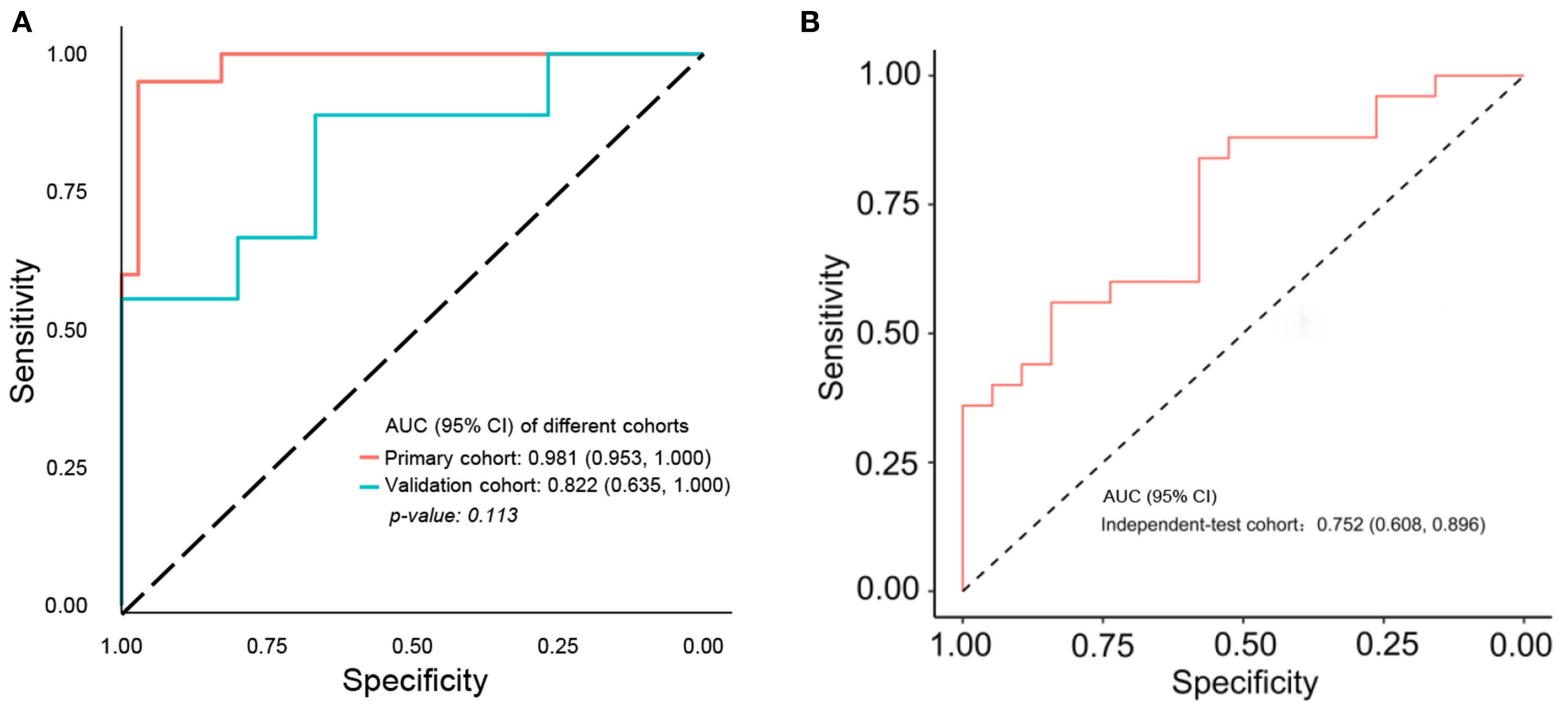
ROC curve of multiscale radiogenomics model. **(A)** ROC curve of multiscale radiogenomics model in the primary cohort and validation cohorts. **(B)** ROC curve of multiscale radiogenomics model in the independent-test cohort.

In order to improve the performance of the model, we combined multidimensional data. Genomics features and clinical risk factors were screened for the construction of the multiscale radiogenomics model. The analysis of RNA sequencing results revealed that the cell cycle pathway was significantly different between the metastasis group and the non-metastasis group (Figures 3A,B). The four cell cycle–related genes (*CDKN2A, TP53, ATM*, and *MYC*) were identified by protein–protein interaction network. Among them, *CDKN2A* was notably increased in the metastasis group (Figure 3C). Previous studies have shown that CA 19-9 is a commonly used tumor marker in clinical practice (18). Remarkably, the multiscale model, combined radiomics with four cell cycle–related genes and clinical risk factor CA 19-9, had better discriminative capacity than the radiomics model. The AUC of the multiscale model reached 0.981 (95% CI 0.953–1.000) and 0.822 (95% CI 0.635–1.000) in the primary and validation cohorts, respectively (Figure 4A). In addition, as shown in Table 2, the multiscale model had better accuracy for assessing CRC preoperative metastasis (sensitivity: 94.4%, specificity: 94.7%, positive predictive value: 97.1%, negative predictive value: 90.0%). To further validate the generalization of multiscale model, we applied the model in an independent-test cohort. The AUC achieved 0.752 (95% CI 0.608–0.896) in the independent-test cohort (Figure 4B), which suggested that the model had good robustness and generalization.

**Table 2:** Performance of the radiomics model and multiscale radiogenomics model in primary and validation cohorts.

**Item**	**Radiomics model**		**Multiscale radiogenomics model**	
	**Primary cohort**	**Validation cohort**	**Primary cohort**	**Validation cohort**
Sensitivity (95% CI)	0.921 (0.786–0.983)	0.846 (0.546–0.981)	0.944 (0.813–0.993)	0.833 (0.516–0.979)
Specificity (95% CI)	0.875 (0.676–0.973)	0.600 (0.323–0.837)	0.947 (0.740–0.999)	0.538 (0.277–0.848)
PPV (95% CI)	0.921 (0.786–0.983)	0.647 (0.383–0.858)	0.971 (0.851–0.999)	0.667 (0.384–0.882)
NPV (95% CI)	0.875 (0.676–0.973)	0.818 (0.482–0.977)	0.900 (0.683–0.988)	0.778 (0.400–0.972)
AUC (95% CI)	0.945 (0.892–0.998)	0.754 (0.570–0.938)	0.981 (0.953–1.000)	0.822 (0.635–1.000)

*PPV, positive predictive value; NPV, negative predictive value; AUC, area under the curve*.

### Clinical Use

**Figure 5:**
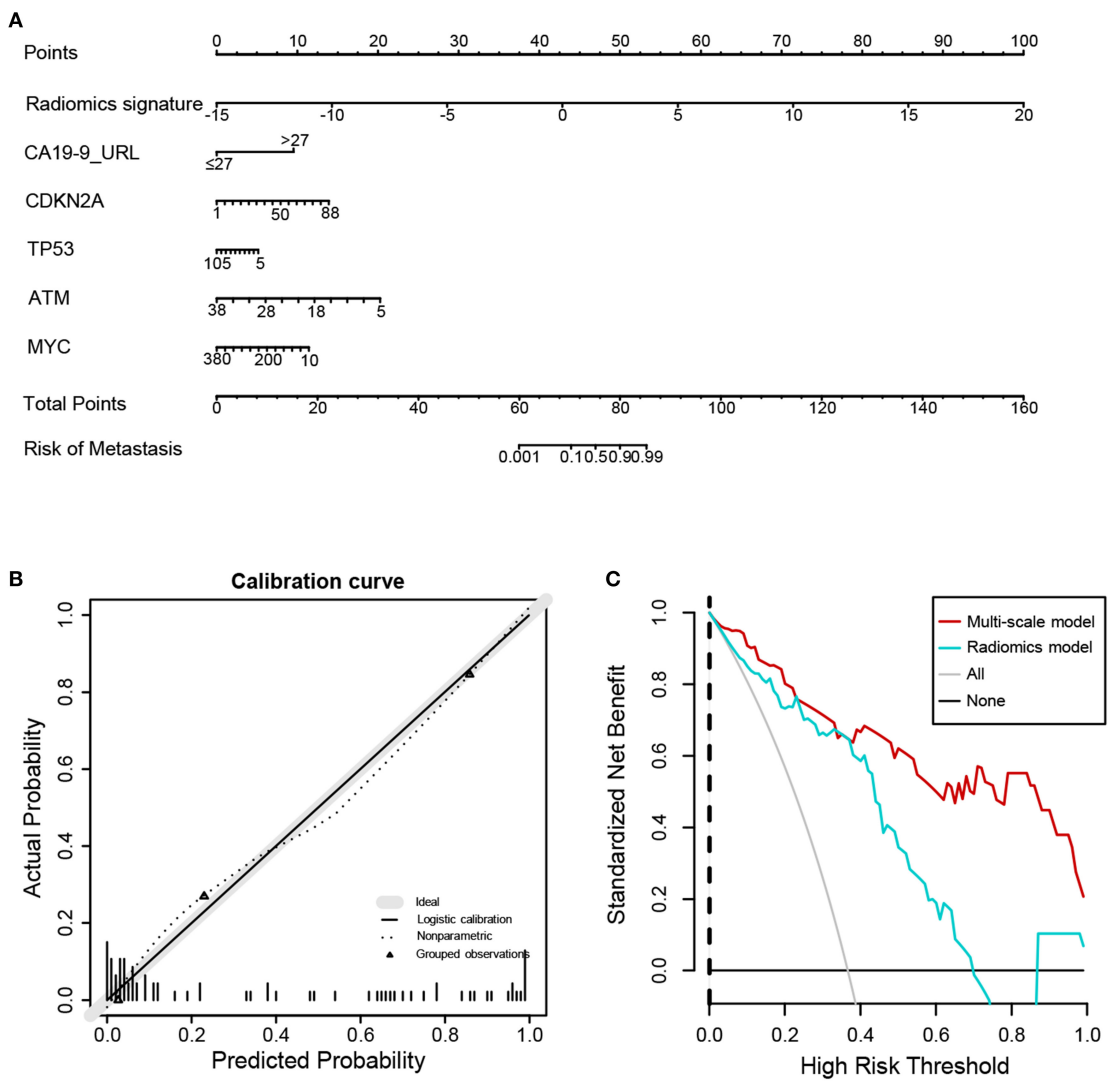
Nomogram, calibration curve, and decision curve derived from the multiscale radiogenomics model. **(A)** Nomogram developed with the radiomics score, genomics, and CA-19-9. **(B)** Calibration curve analysis for the multiscale radiogenomics model. The *y* axis represents the actual probability of metastasis. The *x* axis represents the predicted probability of metastasis. **(C)** Decision curve analysis for the multiscale radiogenomics model.

Next, we further constructed the multiscale nomogram for clinical use, based on radiomics feature, genomics characteristics, and CA 19-9 (Figure 5A). Good calibration was observed for the probability of CRC metastasis (Figure 5B). The decision curve analyses of the radiomics model and the multiscale model are shown in Figure 5C. The decision curve illustrated that both models had relatively good performance for clinical application. Importantly, the multiscale model indicated higher benefit than the radiomics model, which suggested that the multiscale model was a reliable clinical utility for assessment of preoperative metastasis in CRC patients.

## Discussion

In this study, we established and validated a multiscale model for individualized preoperative metastasis assessment in patients with CRC. The multiscale nomogram incorporated radiomics features, genomics signatures, and clinical risk factor. The convenient multiscale nomogram facilitated the individualized evaluation of CRC preoperative metastasis.

Metastasis is the leading cause of reduced 5-years survival rate for patients with CRC (2). Accurate evaluation of metastasis is critical for optimizing the CRC treatment strategies. Currently, the discrimination of CRC metastasis based on the visual judgment of clinicians remains challenging. Quantitative high-throughput radiomics analysis provides many high-dimensional imaging characteristics based on medical imaging, and the main applications are diagnosis, treatment planning, and evaluations in the field of oncology (29). Previous studies have demonstrated that radiomics is used successfully in the prediction of lymph node metastasis and of outcomes (19, 30). Moreover, the combined analysis using multiple markers performs better than individual analysis in clinical practice (19, 31, 32). Meanwhile, risk stratification and clinical decision-making are better to assess the disease risk by polygenic risk score (33). Exploring the genomics of cancer patients might help to optimize therapeutic strategies. Previous study has proposed that the gene expression could be evaluated by radiomics features in CRC (34). However, the potential associations between radiomics, gene expression, and clinical risk remain unclear.

In this study, the regions of interest on enhanced venous-phase CT were delineated to screen for metastasis-related image features. Eight hundred fifty-four radiomics features, extracted from enhanced venous-phase CT, were reduced to 16 potential predictors by LASSO. The AUCs of the radiomics model were 0.945 (95% CI 0.892–0.998) and 0.754 (95% CI 0.570–0.938) in the primary and validation cohorts, respectively. To enhance the performance of the model, we combined gene expression and clinical risk factors. The 90 CRC tissues were analyzed by RNA sequencing, and we selected four genes (*CDKN2A, TP53, ATM*, and *MYC*) closely related to the cell cycle based on the RNA sequencing analysis. Existing studies have shown that *CDKN2A, TP53, ATM*, and *MYC* play an important role in progression and metastasis of CRC diseases (35–38). Our study indicated that these four genes were identified as genomics risk features for assessment of CRC metastatic status. Previous studies have shown that CA 19-9 could be used as an important indicator for the prognosis of patients with CRC (39). Moreover, it has been demonstrated that CA 19-9 could be used to monitor the disease development in the metastatic CRC patients without of CEA elevation (40). Therefore, CA 19-9 was kept as a predictor during the process of model establishment. In this study, we combined genomics features and CA 19-9 with radiomics signatures to develop a multiscale model for evaluating the metastasis of CRC. The AUC of the multiscale model [0.981 (95% CI 0.953–1.000) and 0.822 (95% CI 0.635–1.000) in the primary and validation cohorts, respectively] was higher than the radiomics model, indicating that the multiscale model had a better performance. These results give us some hints, that is, while focusing on radiomics, the convergence of multiple omics will be more valuable for disease prediction and diagnosis.

We utilized a nomogram as an individualized tool for CRC preoperative metastasis detection and evaluated whether the multiscale nomogram on the basis of decisions could benefit patients. Decision curve analysis was performed for estimating the clinical consequences of the multiscale nomogram based on threshold probability. The decision curves indicated that the threshold probability ranged from 0 to 100%, implying that applying the multiscale nomogram to assess CRC metastatic status adds more net benefit than the “treat-all” or “treat-none” scheme. As expected, the net benefit of the multiscale model was better than the radiomics model, suggesting that the multiscale model could preferably assist both clinicians and patients to evaluate the risk of CRC preoperative metastasis.

There are several limitations to our study that deserve recognition. First, this is a single-center study with limited generalizability. Second, the sample size of the study is relatively small. Third, the research period and follow-up time were not long enough; some metastasis may be omitted. A large sample of patients from multiple centers should be studied to improve the robustness and reproducibility of our developed multiscale model.

In conclusion, this study presented a multiscale model that incorporated the radiological eigenvalues, genomics features, and CA 19-9, which could be conveniently utilized to facilitate the individualized evaluation of CRC preoperative metastasis.

## Data Availability Statement

The original contributions presented in the study are pubicly available. This data can be found here: https://bigd.big.ac.cn/gsa-human under accession number HRA000235.

## Ethics Statement

The studies involving human participants were reviewed and approved by the ethics committee of Dazhou Central Hospital. The patients/participants provided their written informed consent to participate in this study.

## Author Contributions

FZ, H-YZ, and JingW participated in the study design and manuscript preparation. QL, JL, LX, and JiaW performed radiomics features extraction. QL, JL, LX, ZZ, JF, XH, and YC performed the samples and clinical data acquisition. QL, JL, JiaW, and FZ analyzed the data. QL, ZZ, FZ, and H-YZ revised the manuscript. All authors contributed to the article and approved the submitted version.

## Conflict of Interest

The authors declare that the research was conducted in the absence of any commercial or financial relationships that could be construed as a potential conflict of interest.

## Supplementary Material

Supplementary Figure 1 Typical cases of non-metastasis and metastasis.

Supplementary Table 1 Characteristics comparison between primary and validation cohorts.

Supplementary Table 2 Characteristic of independent-test cohort.

## Abbreviations

CRC: colorectal cancer
CT: computed tomography
CA 19-9: carbohydrate antigen 19-9
AUC: area under curve
ROC: receiver operating characteristic curve
LASSO: least absolute shrinkage and selection operator
CI: confidence interval
PPV: positive predictive value
NPV: negative predictive value.
