# Supplementary material for: Individualized Prediction of Colorectal Cancer Metastasis Using a Radiogenomics Approach
Source: Front Oncol. 2021 Apr 28;11:620945. doi: 10.3389/fonc.2021.620945 (PMC8113949; doi:10.3389/fonc.2021.620945)
Supplement: Supplementary Figure 1 — Typical cases of non-metastasis and metastasis. [file Data_Sheet_1.PDF]

Case 1. 56 years colorectal cancer patient without metastasis

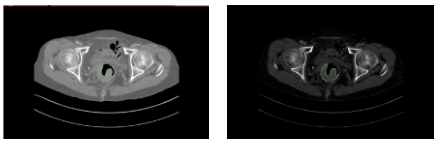

original_shape_Flatness: 0.4588
original_glszm_LargeAreaLowGrayLevelEmphasis: 2026.996
original_glszm_ZoneEntropy: 5.648011
wavelet_HLL_firstorder_Kurtosis: 35.83267
wavelet_LHH_glszm_SmallAreaEmphasis: 0.433857
wavelet_LHH_ngtdm_Coarseness: 6.29E-05
wavelet_LLH_gldm_DependenceNonUniformityNormalized: 0.079423
wavelet_HLH_firstorder_Median: -0.04769
wavelet_HHH_gldm_SmallDependenceLowGrayLevelEmphasis: 0.001548
wavelet_HHH_glszm_ZoneVariance:53317242
……

Case2. 65 years colorectal cancer patient with metastasis.

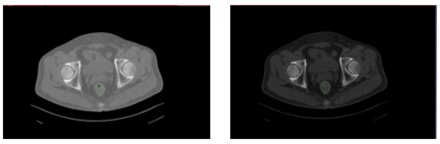

original_shape_Flatness: 0.383702
original_glszm_LargeAreaLowGrayLevelEmphasis: 645.3682
original_glszm_ZoneEntropy: 5.752329
wavelet_HLL_firstorder_Kurtosis: 14.93975
wavelet_LHH_glszm_SmallAreaEmphasis: 0.456977
wavelet_LHH_ngtdm_Coarseness: 0.00017
wavelet_LLH_gldm_DependenceNonUniformityNormalized: 0.103084
wavelet_HLH_firstorder_Median: 0.194586
wavelet_HHH_gldm_SmallDependenceLowGrayLevelEmphasis: 0.001328
wavelet_HHH_glszm_ZoneVariance: 59353493
……

Figure S1. Typical cases of non-metastasis and metastasis.

Table S1. Characteristics comparison between primary and validation cohorts.

Characteristic	Primary cohort	Validation cohort	*P*
Age (mean±SD)	59.21±11.60	60.68±12.16	0.585
Sex, No. (%)			0.710
Male	38 (61.29)	16 (57.14)	
Female	24 (38.71)	12 (42.86)	
CA19-9 level, No. (%)			0.651
Normal	42 (71.19)	19 (76.00)	
Abnormal	17 (28.81)	6 (24.00)	
Tumor stage, No. (%)			0.209
0	4 (6.45)	0 (0.00)	
I	18 (29.03)	7 (25.00)	
II	16 (25.81)	10 (35.71)	
III	20 (32.26)	6 (21.43)	
IV	4 (6.45)	5 (17.86)	
Tumor sites, No. (%)			0.157
Rectum	42 (67.74)	19 (67.86)	
Right colon	8 (12.90)	5 (17.86)	
Left colon	7 (11.29)	1 (3.57)	
Sigmoid colon	5 (8.06)	1 (3.57)	
Transverse colon	0 (0.00)	2 (7.14)	
Radiomics score (mean±SD)	-0.97±5.53	1.14±5.61	0.099

Note: T test or Chi-square test was used to compare characteristics differences between primary and validation cohorts. The upper reference limit value for CA19-9 level was 27 ng/mL in clinic. Abnormal：CA19-9 level >27 ng/mL; Normal：CA19-9 level ≤ 27 ng/mL.
Abbreviations: SD, standard deviation; CA19-9, carbohydrate antigen 19-9.

Table S2. Characteristic of independent-test cohort.

Characteristic	Independent-test cohort		*P*
	Non-metastasis （n=19）	Metastasis （n=25）	
Age (mean±SD)	59.74±9.90	61.80±14.55	0.598
Sex, No. (%)			0.328
Male	11 (57.89)	18 (72.00)	
Female	8 (42.11)	7 (28.00)	
CA19-9 level, No. (%)			0.773
Normal	17 (89.47)	23 (92.00)	
Abnormal	2 (10.53)	2 (8.00)	
Tumor stage, No. (%)			< 0.001*
0	0 (0.00)	0 (0.00)	
I	11 (57.89)	0 (0.00)	
II	8 (42.11)	0 (0.00)	
III	0 (0.00)	23 (92.00)	
IV	0 (0.00)	2 (8.00)	
Tumor sites, No. (%)			0.364
Rectum	14 (73.68)	20 (80.00)	
Right colon	2 (10.53)	0 (0.00)	
Left colon	0 (0.00)	1 (4.00)	
Sigmoid colon	3 (15.79)	3 (12.00)	
Transverse colon	0 (0.00)	1 (4.00)	

Note: T test or Chi-square test was used to compare characteristics differences between primary and validation cohorts. The upper reference limit value for CA19-9 level was 27 ng/mL in clinic. Abnormal：CA19-9 level >27 ng/mL; Normal：CA19-9 level ≤ 27 ng/mL.
Abbreviations: SD, standard deviation; CA19-9, carbohydrate antigen 19-9.

Supplementary File 1

Radiomics score calculation formula:

Radiomics score = -0.3130-0.0390 ×original_shape_Flatness

-0.6710 × original_glszm_LargeAreaLowGrayLevelEmphasis

-3.6921 × original_glszm_ZoneEntropy

+1.8634 × wavelet_HLL_firstorder_Kurtosis

+0.0415 × wavelet_LHH_glszm_SmallAreaEmphasis

-1.9896 × wavelet_LHH_ngtdm_Coarseness

-0.6571 × wavelet_LLH_gldm_DependenceNonUniformityNormalized

+1.3057 × wavelet_HLH_firstorder_Median

+0.2454 × wavelet_HHH_gldm_SmallDependenceLowGrayLevelEmphasis

+1.0689 × wavelet_HHH_glszm_ZoneVariance

+1.6354 × wavelet_HHH_glszm_SmallAreaLowGrayLevelEmphasis

-3.1844 × wavelet_HHH_ngtdm_Strength

-0.7619 × wavelet_HHL_glszm_GrayLevelNonUniformityNormalized

-6.6192 × wavelet_LLL_firstorder_Median

+3.6785 × wavelet_LLL_firstorder_RootMeanSquared

+1.8980 × wavelet_LLL_ngtdm_Coarseness
